# Supplementary material for: Doenjang Ameliorates Diet-Induced Hyperlipidemia and Hepatic Oxidative Damage by Improving Lipid Metabolism, Oxidative Stress, and Inflammation in ICR Mice
Source: Foods. 2024 May 10;13(10):1471. doi: 10.3390/foods13101471 (PMC11120292; doi:10.3390/foods13101471)
Supplement: Supplementary file 1 [file foods-13-01471-s001.zip › foods-2960176-supplementary.pdf]

SUPPLEMENTARY TABLES

Table S1: Diet composition

	ND	HD	DS
casein	117.5	117.5	105.7163
L-Cystine	2.75	2.75	2.75
corn starch	315	106	91.87596
maltodextrin 10	50	50	50
Sucrose	50	50	50
lard	16.5	110	107.6085
soybean oil	10	10	10
Cholesterol	0	1	1
Cellulose	25	25	25
mineral mix	17.5	17.5	17.5
Calcium Carbonate	15	15	15
Vitamin Mix	5	5	5
Choline bitartrate	1.25	1.25	1.25
된장	0	0	102.2
total(g)	625.5	511	584.9008
kcal	4759	4770	4770
kcal/g	3.804157	4.667319	4.667319

Note: The carbohydrate, protein and fat content of the Doenjang sample was taken into consideration during formulation and were adjusted accordingly to result in similar ratio of all macronutrients.

Table S2: Primer sequence

Gene name	Primer sequence (5′->3′)
β-actin	f- AGC CTT CCT TCT TGG GTA TGG
	r- AGC CTT CCT TCT TGG GTA TGG
CPT-1	f- AAA GAT CAA TCG GAC CCT AGA CA
	r- CAG CGA GTA GCG CAT AGT CA
PPARα	f- GGA TGT CAC ACA ATG CAA TTC GCT
	r- TCA CAG AAC GGC TTC CTC AGG TT
PPARγ	f- GAT CAA AGA GGA GCC AGT GC
	r- GGC CAG CAT CGT GTA GAT GA
SOD	f- CAG GAC CTC ATT TTA ATC CTC AC
	r- TGC CCA GGT CTC CAA CAT
CAT	f- CCT TCA AGT TGG TTA ATG CAG A
	r- CAA GTT TTT GAT GCC CTG GT
NRF2	f- GCC TCC AAA GGA TGT CAA TCA
	r- GCC TCA CCT CTG CTG CAA GTA
GPx1	f- TTT CCC GTG CAA TCA GTT C
	r- TCG GAC GTA CTT GAG GGA AT
SREBP-1c	f- GAT CAA AGA GGA GCC AGT GC
	r- TAG ATG GTG GCT GCT GAG TG
FAS	f- AGG GGT CGA CCT GGT CCT CA
	r- GCC ATG CCC AGA GGG TGG TT
ACC	f- CCA ACA TGA GGA CTA TAA CTT CCT
	r- TAC ATA CGT GCC GTC AGG CTT CAC
NF-kB	f- GCT GCC AAA GAA GGA CAC GAC A
	r- GGC AGG CTA TTG CTC ATC ACA G
IL6	f- TAC CAC TTC ACA AGT CGG AGG C
	r- CTG CAA GTG CAT CAT CGT TGT TC
hmgcr	f- GCT CGT CTA CAG AAA CTC CAC G
	r- GCT TCA GCA GTG CTT TCT CCG T
LXR	f- GTT ATA ACC GGG AAG ACT TTG C
	r- AAA CTC GGC ATC ATT GAG TTG
Glut4	f- GTA ACT TCA TTG TCG GCA TGG
	r- AGC TGA GAT CTG GTC AAA CG
PGC-1α	f- GAA TCA AGC CAC TAC AGA CAC CG
	r- CAT CCC TCT TGA GCC TTT CGT G
G6P	f- GAC CAA GAA GCC TGG CAT GTT C
	r- AGA CAT CCA GGA TGA GGC GTT C
